# Perioperative Adverse Events in Primary Palatoplasty: “Risk Factors and Impact on Care Escalation in a Multicenter Cohort”

**DOI:** 10.1002/pan.70224

**Published:** 2026-05-22

**Authors:** Febina Padiyath, Elizabeth M. O'Brien, Jennifer Nam, Paula Hu, Nishil Mehta, Marc Parris, Anthony Alexander, Ashley Hodges, Goretti Chiang, Vinícius Caldeira Quintão, Ricardo Vieira Carlos, Gabriel Soares de Sousa, Asif Padiyath, Susan C. Nicolson, John Fiadjoe, Oksana A. Jackson, Lisa K. Lee, Annery G. Garcia‐Marcinkiewicz

**Affiliations:** ^1^ Department of Anesthesiology and Critical Care Medicine Children's Hospital of Philadelphia Philadelphia Pennsylvania USA; ^2^ Perelman School of Medicine at the University of Pennsylvania Philadelphia Pennsylvania USA; ^3^ Department of Anesthesiology and Perioperative Medicine Ronald Reagan UCLA Medical Center, David Geffen School of Medicine Los Angeles California USA; ^4^ Drexel University College of Medicine Philadelphia Pennsylvania USA; ^5^ Instituto da Criança e do Adolescente, Hospital das Clínicas HCFMUSP, Faculdade de Medicina, Universidade de São Paulo São Paulo Brazil; ^6^ Division of Plastic, Reconstructive and Oral Surgery, Department of Surgery Children's Hospital of Philadelphia Philadelphia Pennsylvania USA

**Keywords:** cleft palate surgery outcomes, hypoxemia and airway obstruction, pediatric airway complications, perioperative adverse events, primary palatoplasty

## Abstract

**Background:**

Patients undergoing palatoplasty experience perioperative adverse events. Identifying risk factors for perioperative adverse events and escalation of care may improve outcomes.

**Methods:**

Pediatric patients undergoing primary palatoplasty were included in this retrospective observational study performed at two academic children's hospitals from 2014 to 2020.

**Results:**

Four hundred fifty‐nine patients with a median age of 12 months (IQR: 10–17) and median weight of 9.3 kg (IQR: 8.3–10.9) were included. A perioperative adverse event occurred in 15% (69/459) patients. Multiple (> 1) perioperative adverse events occurred in 18 patients. Hypoxemia (42 events, 9.2%) was the most common perioperative adverse event, followed by upper airway obstruction (29 events, 6.3%), vomiting (11 events, 2.4%), hypoventilation (10 events, 2.2%), and bleeding (7 events, 1.5%). Two events required a rapid response team, and there was 1 cardiac arrest. Forty percent of perioperative adverse events occurred in the post‐anesthesia recovery area, and 24% occurred within 12 h of arriving to the floor. Prolonged emergence time (OR 1.04 [95% CI 1.02–1.06], *p* < 0.001) and nasal stent placement (OR 6.02 [95% CI 2.7–13.6], *p* < 0.001) were significantly associated with perioperative adverse events and increased odds of unanticipated admission to the pediatric intensive care unit (OR 1.02 [95% CI, 1.01–1.04], *p* < 0.001) and (OR 3.39 [95% CI, 1.19–8.83], *p* = 0.016), respectively. The occurrence of any perioperative adverse event (OR 8.47 [95% CI, 4.06–18], *p* < 0.001) and increasing number of perioperative adverse events (OR 1.75 [95% CI, 1.41–2.24], *p* < 0.001) were also associated with increased odds of unanticipated PICU admission.

**Conclusions:**

Patients undergoing palatoplasty are at risk of perioperative adverse events, particularly in post‐operative recovery areas, which may result in unanticipated escalation of care. Recognizing the risk factors associated with the development of perioperative adverse events may improve planning of post‐operative care and outcomes.

## 1 Introduction

Cleft palate is one of the most common birth defects worldwide [1]. Palatoplasties are performed with the goal of obtaining velopharyngeal competence and normal speech. Repair can improve the function and quality of life of patients; it is typically performed between 9 and 18 months of age [2]. General anesthesia is routinely used for infants and children having cleft palate repair. However, by the very nature of their condition, patients requiring palatoplasty tend to have abnormal airway anatomy and a postoperative decrease in upper airway volume [3, 4]. Perioperative adverse events have been reported in patients undergoing palatoplasty leading to significant morbidity, distress, and increased health care costs [5]. One recent study looking at perioperative adverse events in this population reported an incidence of 23% [6]. The overall incidence of serious respiratory events in healthy pediatric surgical patients with normal airway anatomy is much lower, reported to be approximately 0.03% [7, 8], and in those with difficult airways approximately 20% [9]. With recent innovations in both the technology and techniques used to provide safer pediatric perioperative care, particularly with airway management [10, 11, 12], we designed this study to determine the current rate of perioperative adverse events in patients undergoing palatoplasty as well as risk factors associated with perioperative adverse events. A secondary objective was to determine the association and rate of escalation of care in those who experience perioperative adverse events. Identifying the risk factors could lead to risk stratification and care pathway optimization.

## 2 Methods

### 2.1 Study Design

This is a retrospective cohort study of all patients who had a primary palatoplasty performed between July 2014 and January 2020 in two academic children's hospitals in the United States. This project was approved by both hospitals' Institutional Review Boards prior to data collection. We adhered to the Strengthening the Reporting of Observational Studies in Epidemiology (STROBE) guidelines when writing this manuscript.

### 2.2 Exposure and Outcomes

The main exposure of interest in this study is primary palatoplasty. The main outcome of interest is the occurrence of any perioperative adverse event. A perioperative adverse event is defined as: hypoxemia (oxygen saturation < 90% for longer than 45 s), upper airway obstruction, hypoventilation, airway bleeding, vomiting, concerning for aspiration, need for rapid response team, epistaxis, esophageal intubation, pneumothorax, hypotension, arrythmia, and death. Risk factors of interest explored to determine the association with perioperative adverse events included patient, surgical, and anesthetic factors. Patient factors included age at repair, weight, race, American Society of Anesthesiologists (ASA) physical status, Veau cleft type, history of cleft lip repair, history of airway anatomic abnormality (such as micro/retrognathia, macroglossia, maxillary hypoplasia, nasal airway disease, glossoptosis, tracheomalacia, laryngeal anomaly), history of obstructive sleep apnea, asthma, upper respiratory infection in the 2 weeks prior to surgery, history of prematurity, history of a diagnosed syndrome or history of Pierre Robin Sequence. Surgical factors included technical details such as use of relaxing incisions, nasal floor closure with vomer flaps, type of repair, concurrent procedures, whether the surgeon injected local anesthetic, and whether the surgeon placed a nasopharyngeal (NP) airway at the end of the case. Anesthetic factors included history of difficult airway, anesthetic induction technique (inhalational or intravenous), use of neuromuscular blocking and reversal agents, and time to emergence from anesthesia (time the anesthesiologist considered as the emergence until extubation). Escalation of care was defined as a patient who was planned to have floor level care post‐operatively who then required an unplanned ICU admission.

### 2.3 Inclusion and Exclusion Criteria

We included all patients who underwent a primary palatoplasty at the Children's Hospital of Philadelphia (CHOP) and the University of California, Los Angeles (UCLA) between July 2014 and January 2020. Patients with significant cardiac disease who required care by a separate cardiac anesthesia team, or if the surgeon performed fewer than ten palatoplasties annually, were excluded. All patients that met the above inclusion criteria were entered into the Cleft Palate Registry at CHOP.

### 2.4 Data Collection

Data were obtained from the medical record and entered into Redcap by trained research assistants.

### 2.5 Statistical Analysis

Categorical variables are reported as proportions and continuous variables as median with interquartile range (IQR). Logistic regression was used to determine the association between perioperative adverse events and variables of interest. *p*‐Value less than 0.05 was deemed statistically significant. Analyses were performed using STATA 15.0 (Stata Corp).

## 3 Results

### 3.1 Patient Characteristics

From July 2014 to January 2020, 464 patients underwent a primary palatoplasty between the two institutions. Five patients were excluded due to missing data on the primary outcome for a total of 459 patients in the study. The median patient age was 12 months (IQR: 10–17), and median weight of 9.3 kg (IQR: 8.3–10.9). Cleft distribution included submucous 12% (53/459), Veau I 17% (80/459), Veau II 35.1% (162/459), Veau III 24% (111/459), and Veau IV 10.6% (49/459). Twenty‐four percent of patients had a diagnosed syndrome with 22q11 deletion being the most frequent in 25% of patients with a syndrome. Patient demographics are summarized in Table 1.

**TABLE 1:** Patient demographics and perioperative characteristics.

Patient demographics	No complications	At least one complication	*p* ^b^
	*N* = 390^a^	*N* = 69^a^	
Age (months)	11 [10‐ 15]	12 [10‐ 18]	
Sex			
Male	205 (52.6%)	42 (60.9%)	
Weight (kg)	9.3 [8.3‐ 10.9]	9.3 [8.5‐ 10.1]	
ASA Physical Status			
ASA 1	45 (11.5%)	8 (11.6%)	
ASA 2	251 (64%)	29 (42%)	
ASA 3	93 (23.8%)	30 (43.5%)	
ASA 4	1 (0.3%)	2 (2.9%)	
Difficult facemask	1 (0.3%)	2 (2.9%)	0.014*
Use of neuromuscular blocking agent	298 (76.4%)	45 (65.2%)	0.048*
Suprazygomatic block	53 (13.59%)	10 (10.42%)	0.841
Local infiltration	146 (38.22%)	16 (24.24%)	0.029*
Upper respiratory infection	86 (22.3%)	18 (26.9%)	0.51
Emergence time	27 [22‐ 35.8]	36 [26.8‐ 47.5]	< 0.001*
Anesthesia stop time	11.9 [10.5‐ 14.5]	13 [11.3‐ 15.8]	0.003*
Veau type			0.35
Submucosal cleft palate	45 (11.7%)	8 (11.6%)	
Type I, soft palate only	66 (17.1%)	14 (20.3%)	
Type II, soft palate/hard palate	132 (34.2%)	30 (43.5%)	
Type III, unilateral cleft lip and palate	100 (25.9%)	11 (15.9%)	
Type IV, bilateral cleft lip and palate	43 (11.1%)	6 (8.7%)	
Nasal stent	20 (5.2%)	15 (21.7%)	< 0.001*
Upgrade in care	20 (5.2%)	23 (33.3%)	< 0.001*

^a^ *n* (%) categorical variables; median [interquartile range] continuous variables.

^b^ Chi‐square test.

* Statistically significant.

### 3.2 Incidence and Types of Perioperative Adverse Events

A single perioperative adverse event occurred in 15% (69/459) patients and multiple (> 1) perioperative adverse events occurred in 3.9% (18/459) patients. Hypoxemia (42 events, 9.2%) was the most common, followed by upper airway obstruction (29 events, 6.3%), vomiting concerning for risk of aspiration (11 events, 2.4%), hypoventilation (10 events, 2.2%), and bleeding (7 events, 1.5%). There were 2 events requiring rapid response team, and 1 cardiac arrest. Data on accidental extubation, need for OR takeback, airway trauma, arrhythmias, epistaxis, esophageal intubation, pneumothorax, and death were collected, but no patient was documented to have these outcomes in this study.

Table 2 demonstrates patient factors associated with perioperative adverse events. American Society of Anesthesiologists (ASA) physical status > 2 (OR 2.68 [95% CI, 1.6–4.71], *p* < 0.001); other anatomic airway abnormality (OR 2.0 [95% CI 1.01–3.63], *p* = 0.04); history of OSA (OR 2.2 [95% CI 1.18–4.16], *p* = 0.01); presence of a syndrome (OR 2.44 [95% CI, 1.36–4.37], *p* = 0.002) were associated with significantly higher odds of having a perioperative adverse event. 73.42% of patients received non‐depolarizing neuromuscular blocking agents. There were fewer complications in patients who received neuromuscular blocking agents (*p*=0.048).

**TABLE 2:** Complication types and rates among patients who had an unanticipated PICU admission compared to patients who experienced a postoperative complication but did not require PICU admission.

	Total (*n* = 459)		Events among patients without unanticipated PICU admission (*n* = 43)		Events among patients with unanticipated PICU admission (*n* = 26)	
	Events	%	Events	%	Events	%
Hypoxia (sat < 90% for more than 45 s)	42	9.2	23	53	19	73
Obstruction	29	6.3	15	35	14	54
Other	27	5.9	12	28	15	58
Vomiting	11	2.4	9	21	2	7.7
Hypoventilation	10	2.2	5	12	5	19
Bleeding	7	1.5	4	9.3	3	12
Broncho/Laryngospasm	5	1.1	2	4.7	3	12
Re‐intubation	3	0.65	1	2.3	2	7.7
Rapid response	3	0.65	2	4.7	1	3.8
Hypotension	2	0.44	1	2.3	1	3.9
Cardiac Arrest	1	0.22	0	0	1	3.9

**FIGURE 1:**
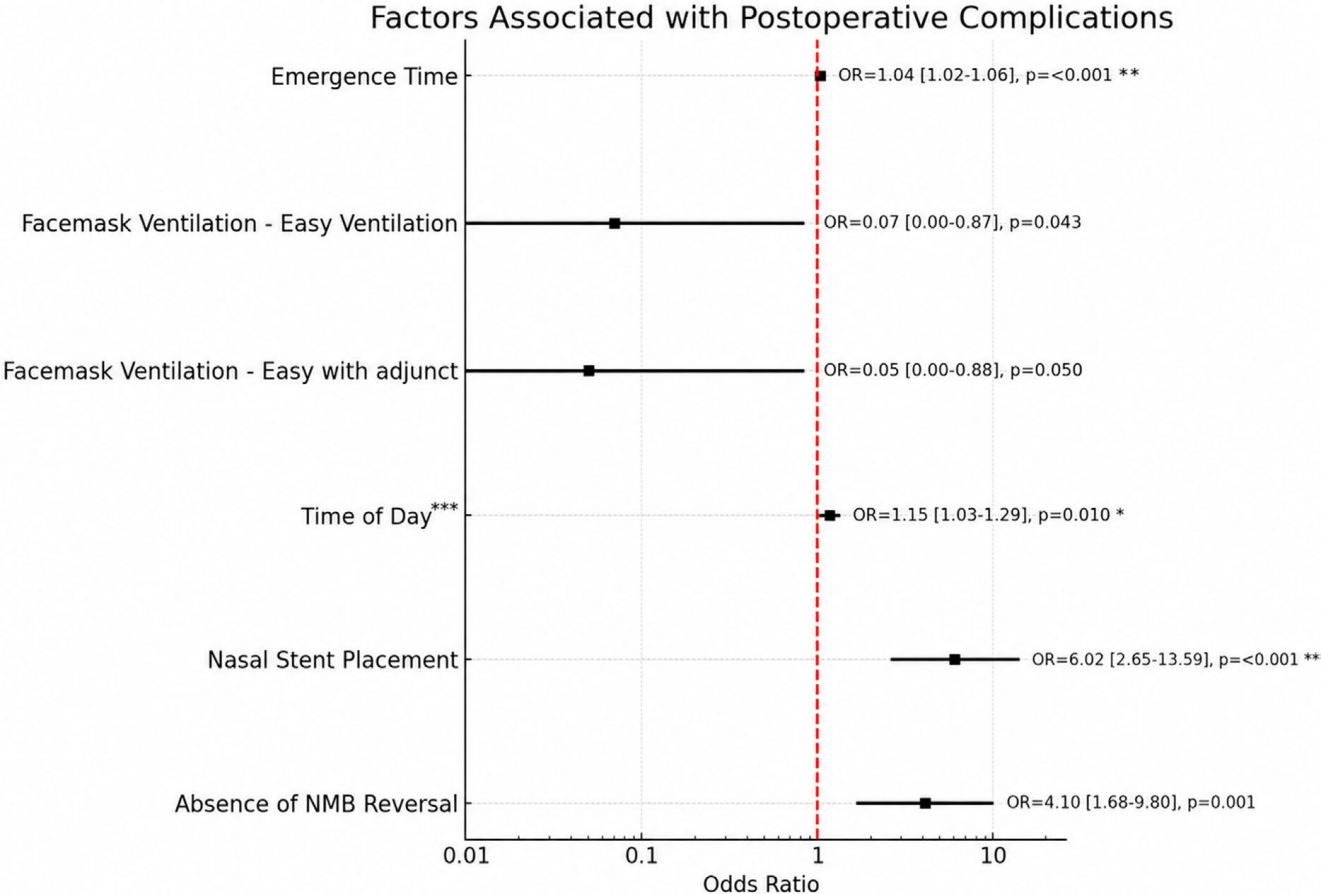
Factors associated with postoperative complications. *Statistically significant after Benjamini‐Hochberg procedure. **Statistically significant after Bonferroni correction. ***Time of Day: Cases occurring later during the work shift have higher odds of complications.

**FIGURE 2:**
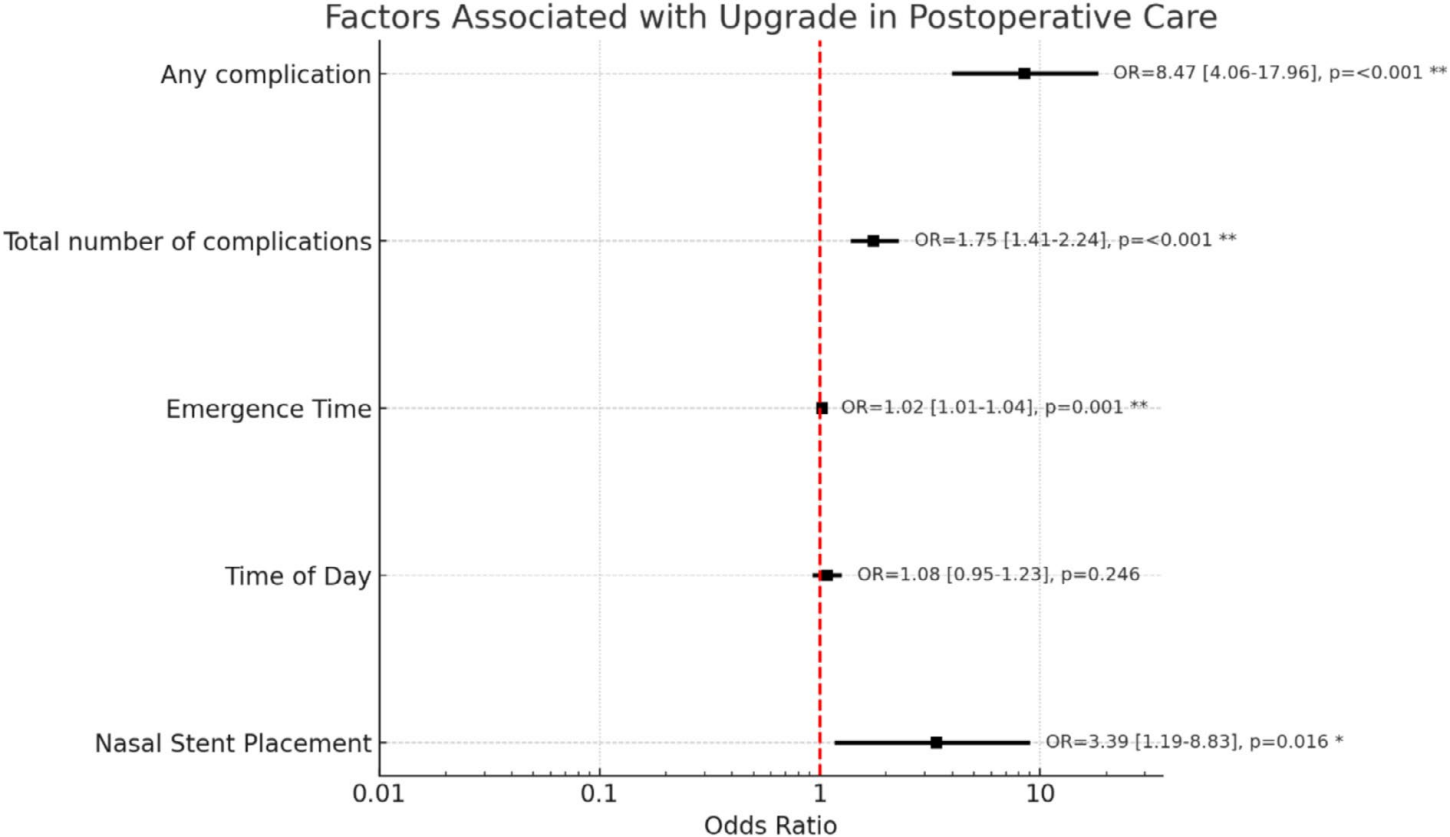
Factors associated with escalation of postoperative care. *Statistically significant after Benjamini‐Hochberg procedure. **Statistically significant after Bonferroni correction.

Surgical and anesthetic factors associated with perioperative adverse events are shown in Figures 1 and 2. Significant surgical factors included the requirement for an NP airway at the end of case to maintain airway patency (OR 2.44 [95% CI, 1.36–4.37], *p* = 0.036). Associated anesthetic factors include the history of difficult airway (OR 2.64 [95% CI, 1.39–5], *p* = 0.002); lack of neuromuscular blockade reversal administration (OR 4.1 [95% CI, 1.68–9.8], *p* < 0.001) and prolonged emergence time (OR 1.03 [95% CI, 1.02–1.04], *p* < 0.0001).

The majority of perioperative adverse events occurred while the patient was in the post‐anesthesia recovery area (PACU), and 24% occurred within 12 h of arriving to the floor post‐operatively. Escalation of care occurred in 5.7% (26/459) of patients. Both the occurrence of any perioperative adverse events (OR 8.47 [95% CI 4.06–17.96], *p* < 0.001) and increasing number of perioperative adverse events (OR 1.75 [95% CI 1.41–2.24], *p* < 0.001) in the postoperative period were also associated with increased odds of unanticipated PICU admission.

## 4 Discussion

In this study we describe the contemporary perioperative outcomes of 459 patients undergoing primary palatoplasty and found the rate of PAEs to be 15%. Pediatric perioperative adverse events can be challenging to study due to varying definitions across institutions, low event rate, and incomplete data capture [13]. Nevertheless, our definition of perioperative adverse events is similar to other published studies [9, 13, 14, 15] and a complication rate of 15% that we report is much higher than major registries even though Basta et al. described a perioperative adverse event rate of 23% in 2018 [6, 7, 15]. The most frequently observed perioperative adverse event was hypoxemia, followed by functional obstructive airway issues and hypoventilation. Although hypoxemia may be regarded as a commonly remediable condition when managing pediatric physiology, every effort must be taken to prevent it. Hypoxemia combined with other non‐severe perioperative adverse events may lead to severe complications like cardiac arrest, one event of which was observed in our study.

Risk factors observed in our cohort of patients associated with perioperative adverse events included ASA status > 2, notable anatomic airway abnormalities on physical exam, history of OSA, history of difficult airway, NP airway requirement at the end of the case by surgeon, lack of reversal of neuromuscular blocking drugs, and prolonged emergence time. Patients presenting for palatoplasty have by definition an anatomic defect involving their palate, oftentimes with associated syndromes or other craniofacial anomalies [16] that can make airway management challenging. It is important to prepare for these increased risks by formulating a plan utilizing devices and techniques that provide the greatest airway management safety for these patients. We have learned from previous work that patients with difficult airways have high complication rates [9, 11, 17]. A patient requiring a NP airway at the end of the case may be indicative of their potential tendency for upper airway obstruction and need for a higher level of observation. Neuromuscular blocking drugs are frequently used to create optimal tracheal intubation and surgical conditions. They have been associated with a reduced risk of tracheal intubation adverse events [18, 19] even in patients with known difficult airways [11]. However, postoperative residual neuromuscular blockade is a known and preventable risk factor for hypoventilation and airway obstruction [20], thus must be fully reversed. Prolonged emergence time is another observed risk factor for PAE in our study. It is possible that patients with prolonged emergence time may require more careful postoperative observation and an assessment if tracheal extubation at the conclusion of the case is appropriate. Indeed, the retrospective nature of this study precluded the analysis of more granular aspects of anesthetic management although there may be additional contributors to the generation of these adverse events.

Escalation of care occurred in 5.7% of our patients and those with perioperative adverse events were more likely to require escalation of care and intensive care admission. In the adult population, Haller and colleagues validated unplanned postoperative ICU admissions as a measure of patient safety and demonstrated that unplanned ICU admissions were associated with a 2‐to‐3 fold increase in 30‐day mortality and increased length of stay in the hospital [21, 22]. Consistent with our findings, studies looking at factors associated with unplanned ICU admissions in pediatric patients found that younger (infants) and more complex patients ASA > 2 are at increased risk [23, 24]. Consistently, airway anomalies and intraoperative hypoxemia have also been found to be associated risks [25]. Unplanned PICU admission poses significant distress to families in addition to increased morbidity, length of stay, and cost of care [26].

### 4.1 Limitations

There are several limitations to our study. Although this is a multicenter study, the data were obtained from only two large pediatric tertiary care institutions. While both centers may have broadly similar perioperative management protocols (e.g., the practice at the time was for nearly all patients to have awake extubation performed), there might still be institution specific variations in anesthetic management, and surgical practices‐such as differences in palate repair techniques, surgical duration, or clinical care pathways for perioperative care and escalation of level of care if clinical deterioration were to occur, which would influence outcomes. At the time the study was performed, most patients were maintained on inhalational anesthetic agents for their case. Very few patients were maintained on total intravenous anesthesia, and therefore, we were unable to investigate the impact of these factors in the present study. Given the retrospective nature of this study, there is potential for selection bias. It is possible that there was limited or lack of data capture on outcomes such as esophageal intubation, epistaxis, accidental extubation. Due to the possibility of this underreporting of events the complications in these patients might be greater than what we observed. As such, our results should be interpreted with caution. A prospective multi‐center study would help address these limitations more comprehensively and allow for standardized data collection across sites prospectively.

## 5 Conclusion

Our data suggests that patients undergoing primary palatoplasty are at risk of perioperative adverse events, particularly in post‐operative recovery areas, which may result in the escalation of care. Recognizing the risk factors associated with the development of perioperative adverse events may improve planning of post‐operative care and outcomes.

## Funding

The authors have nothing to report.

## Conflicts of Interest

The authors declare no conflicts of interest.

## Data Availability

The data that support the findings of this study are available on request from the corresponding author. The data are not publicly available due to privacy or ethical restrictions.

## References

[1] S. Thaller , J. Bradley , and J. Garri , Craniofacial Surgery (Informa Healthcare, 2008).

[2] F. P. Cladis , L. Grunwaldt , and J. Losee , “Anesthesia for Pediatric Plastic Surgery,” in Smith's Anesthesia for Infants and Children, 9th ed. (Elsevier, 2017).

[3] M. Bacher , U. Bacher , G. Goz , et al., “Three‐Dimensional Computer Morphometry of the Maxilla and Face in Infants With Pierre Robin Sequence—A Comparative Study,” Cleft Palate‐Craniofacial Journal 37, no. 3 (2000): 292–302, 10.1597/1545-1569_2000_037_0292_tdcmot_2.3.co_2. 10830810

[4] C. Stransky , M. Basta , C. Solot , et al., “Do Patients With Pierre Robin Sequence Have Worse Outcomes After Cleft Palate Surgery?,” Annals of Plastic Surgery 71, no. 3 (2013): 292–296, 10.1097/SAP.0b013e3182898712. 23676521

[5] J. A. Owusu , M. Liu , J. D. Sidman , and A. R. Scott , “Resource Utilization in Primary Repair of Cleft Palate,” Laryngoscope 123, no. 3 (2013): 787–792, 10.1002/lary.23661. 23070822

[6] M. N. Basta , J. E. Fiadjoe , A. S. Woo , K. N. Peeples , and O. A. Jackson , “Predicting Adverse Perioperative Events in Patients Undergoing Primary Cleft Palate Repair,” Cleft Palate‐Craniofacial Journal 55, no. 4 (2018): 574–581, 10.1177/1055665617744065. 29554444

[7] M. Hache , L. S. Sun , G. Gadi , et al., “Outcomes From Wake Up Safe, the Pediatric Anesthesia Quality Improvement Initiative,” Paediatric Anaesthesia 30, no. 12 (2020): 1348–1354, 10.1111/pan.14044. 33078514

[8] W. Habre , N. Disma , K. Virag , et al., “Incidence of Severe Critical Events in Paediatric Anaesthesia (APRICOT): A Prospective Multicentre Observational Study in 261 Hospitals in Europe,” Lancet Respiratory Medicine 5, no. 5 (2017): 412–425, 10.1016/S2213-2600(17)30116-9. 28363725

[9] J. E. Fiadjoe , A. Nishisaki , N. Jagannathan , et al., “Airway Management Complications in Children With Difficult Tracheal Intubation From the Pediatric Difficult Intubation (PeDI) Registry: A Prospective Cohort Analysis,” Lancet Respiratory Medicine 4, no. 1 (2016): 37–48, 10.1016/S2213-2600(15)00508-1. 26705976

[10] A. G. Garcia‐Marcinkiewicz , P. G. Kovatsis , A. I. Hunyady , et al., “First‐Attempt Success Rate of Video Laryngoscopy in Small Infants (VISI): A Multicentre, Randomised Controlled Trial,” Lancet 396, no. 10266 (2020): 1905–1913, 10.1016/S0140-6736(20)32532-0. 33308472

[11] A. G. Garcia‐Marcinkiewicz , H. D. Adams , H. Gurnaney , et al., “A Retrospective Analysis of Neuromuscular Blocking Drug Use and Ventilation Technique on Complications in the Pediatric Difficult Intubation Registry Using Propensity Score Matching,” Anesthesia and Analgesia 131, no. 2 (2020): 469–479, 10.1213/ANE.0000000000004393. 31567318

[12] J. Peyton , R. Park , S. J. Staffa , et al., “A Comparison of Videolaryngoscopy Using Standard Blades or Non‐Standard Blades in Children in the Paediatric Difficult Intubation Registry,” British Journal of Anaesthesia 126, no. 1 (2021): 331–339, 10.1016/j.bja.2020.08.010. 32950248

[13] L. E. Schleelein , A. M. Vincent , A. F. Jawad , et al., “Pediatric Perioperative Adverse Events Requiring Rapid Response: A Retrospective Case‐Control Study,” Paediatric Anaesthesia 26, no. 7 (2016): 734–741, 10.1111/pan.12922. 27198531

[14] A. Nishisaki , D. A. Turner , C. A. Brown, 3rd , et al., “A National Emergency Airway Registry for Children: Landscape of Tracheal Intubation in 15 PICUs,” Critical Care Medicine 41, no. 3 (2013): 874–885, 10.1097/CCM.0b013e3182746736. 23328260

[15] A. L. Graciano , R. Tamburro , A. E. Thompson , J. Fiadjoe , V. M. Nadkarni , and A. Nishisaki , “Incidence and Associated Factors of Difficult Tracheal Intubations in Pediatric ICUs: A Report From National Emergency Airway Registry for Children: NEAR4KIDS,” Intensive Care Medicine 40, no. 11 (2014): 1659–1669, 10.1007/s00134-014-3407-4. 25160031

[16] A. G. Garcia‐Marcinkiewicz and P. A. Stricker , “Craniofacial Surgery and Specific Airway Problems,” Paediatric Anaesthesia 30, no. 3 (2020): 296–303, 10.1111/pan.13790. 31837242

[17] N. E. Burjek , A. Nishisaki , J. E. Fiadjoe , et al., “Videolaryngoscopy Versus Fiber‐Optic Intubation Through a Supraglottic Airway in Children With a Difficult Airway: An Analysis From the Multicenter Pediatric Difficult Intubation Registry,” Anesthesiology 127, no. 3 (2017): 432–440, 10.1097/ALN.0000000000001758. 28650415

[18] C. Mamie , W. Habre , C. Delhumeau , C. B. Argiroffo , and A. Morabia , “Incidence and Risk Factors of Perioperative Respiratory Adverse Events in Children Undergoing Elective Surgery,” Paediatric Anaesthesia 14, no. 3 (2004): 218–224, 10.1111/j.1460-9592.2004.01169.x. 14996260

[19] L. H. Lundstrom , C. H. V. Duez , A. K. Norskov , et al., “Effects of Avoidance or Use of Neuromuscular Blocking Agents on Outcomes in Tracheal Intubation: A Cochrane Systematic Review,” British Journal of Anaesthesia 120, no. 6 (2018): 1381–1393, 10.1016/j.bja.2017.11.106. 29793603

[20] L. P. Fortier , D. McKeen , K. Turner , et al., “The RECITE Study: A Canadian Prospective, Multicenter Study of the Incidence and Severity of Residual Neuromuscular Blockade,” Anesthesia and Analgesia 121, no. 2 (2015): 366–372, 10.1213/ANE.0000000000000757. 25902322

[21] G. Haller , P. S. Myles , R. Wolfe , A. M. Weeks , J. Stoelwinder , and J. McNeil , “Validity of Unplanned Admission to an Intensive Care Unit as a Measure of Patient Safety in Surgical Patients,” Anesthesiology 103, no. 6 (2005): 1121–1129, 10.1097/00000542-200512000-00004. 16306722

[22] G. Haller , P. S. Myles , M. Langley , J. Stoelwinder , and J. McNeil , “Assessment of an Unplanned Admission to the Intensive Care Unit as a Global Safety Indicator in Surgical Patients,” Anaesthesia and Intensive Care 36, no. 2 (2008): 190–200, 10.1177/0310057X0803600209. 18361010

[23] E. K. Landry , R. A. Gabriel , S. Beutler , R. P. Dutton , and R. D. Urman , “Analysis of Unplanned Intensive Care Unit Admissions in Postoperative Pediatric Patients,” Journal of Intensive Care Medicine 32, no. 3 (2017): 204–211, 10.1177/0885066616661152. 27530513

[24] J. Mitchell , S. Clement de Clety , E. Collard , et al., “Unplanned Intensive Care Unit Admission After General Anaesthesia in Children: A Single Centre Retrospective Analysis,” Anaesthesia, Critical Care & Pain Medicine 35, no. 3 (2016): 203–208, 10.1016/j.accpm.2015.10.005. 26804922

[25] P. S. da Silva , V. E. de Aguiar , and M. C. Fonseca , “Risk Factors and Outcomes of Unplanned PICU Postoperative Admissions: A Nested Case‐Control Study,” Pediatric Critical Care Medicine 14, no. 4 (2013): 420–428, 10.1097/PCC.0b013e3182720fab. 23439460

[26] M. E. Clark , B. M. Cummings , K. Kuhlthau , N. Frassica , and N. Noviski , “Impact of Pediatric Intensive Care Unit Admission on Family Financial Status and Productivity: A Pilot Study,” Journal of Intensive Care Medicine 34, no. 11–12 (2019): 973–977, 10.1177/0885066617723278. 28797189

